# User-centred design and evaluation of an mHealth app for fathers’ perinatal mental health: a feasibility, acceptability, and usability study

**DOI:** 10.1080/0144929X.2025.2502474

**Published:** 2025-05-09

**Authors:** Samantha J. Teague, Adrian B. R. Shatte, Matthew Fuller-Tyszkiewicz, Delyse M. Hutchinson

**Affiliations:** a SEED Lifespan Strategic Research Centre, School of Psychology, Deakin University, Burwood, Australia; b Department of Psychology, College of Healthcare Sciences, Department of Psychology, College of Healthcare Sciences, James Cook University, Townsville, Australia; c Department of Information Technology, College of Science and Engineering, James Cook University, Townsville, Australia; d Centre for Adolescent Health, Murdoch Children's Research Institute, Royal Children's Hospital, Melbourne, Australia; e Department of Paediatrics, University of Melbourne, Melbourne, Australia; f National Drug and Alcohol Research Centre, University of New South Wales, Sydney, Australia

**Keywords:** Fathers, mHealth, digital mental health intervention, feasibility study, user-centred design

## Abstract

Fathers’ perinatal mental health is a major public health issue, yet few interventions have been developed targeting this group. Fathers face many barriers in accessing perinatal mental health support, including stigma around caregiving and mental health, and thus require careful consideration when designing interventions. This study aimed to examine the feasibility, acceptability, and usability of a mobile app-based intervention for paternal perinatal depression, anxiety, and stress. Following a design science approach, five meta design principles and 15 specific principles were created to guide the intervention design, and a prototype app titled Rover was created. The prototype was evaluated by 43 fathers and 10 mental health clinicians. Participants in both groups rated the app highly for its functionality, clinical content, aesthetics, and digital therapeutic alliance. Qualitative feedback indicated that fathers held particularly favourable views regarding the mood tracking, mindfulness, and goal tracking features. Both groups expressed a preference for more support for the personalisation of content, including more dynamic interactions with the chatbot support feature. To our knowledge, this is the first app-based mental health intervention designed specifically for fathers, with study results providing guidance to the field on developing digital health initiatives for this population.

## 1. Introduction

Perinatal mental health is a significant public health concern in both mothers and fathers alike; however, comparatively less research has been conducted on ways to reach and engage fathers (Bateson et al. 2017; Reay et al. 2023; Tully et al. 2018; Wong et al. 2016). Prevalence rates suggest that approximately 10% of men in the UK, US and Australia experience postpartum depression and up to 18% experience postpartum anxiety (Leach et al. 2016; Paulson and Bazemore 2010). Beyond the core symptoms of depression and anxiety, fathers with postpartum depression and anxiety also exhibit higher rates of anger, substance use, job dissatisfaction, and poor physical health (Goldstein et al. 2020). These factors impact mothers’ wellbeing directly through antisocial behaviours and lack of emotional and parenting support, as well as indirectly through reduced income and housing (Fisher et al. 2021). Inter-parent risk factors have been consistently identified for fathers’ perinatal depression and anxiety, including mothers’ perinatal mental health, lack of social support, and relationship dissatisfaction (Anding et al. 2016; Chhabra, McDermott, and Li 2020; Serhan et al. 2013). Further, fathers’ postpartum depression and anxiety has a specific and persistent impact on their children’s social, emotional, cognitive, and behavioural development, similar in magnitude to that due to maternal postpartum depression and anxiety (Gutierrez-Galve et al. 2019; Ramchandani and Psychogiou 2009). Importantly, emerging research suggests that solely treating maternal postpartum depression and anxiety may not satisfactorily resolve child risk of behavioural and emotional problems due to untreated paternal postpartum depression and anxiety (Fisher et al. 2021).

Research on effective interventions for fathers’ perinatal mental health is scarce, highlighting a need for high-quality, evidence-based inquiry (Goldstein et al. 2020; O’Brien et al. 2017). A recent review identified only 10 interventions addressing fathers’ perinatal depression and anxiety evaluated in the past 25 years, none of which targeted fathers’ perinatal mental health as its primary objective (Goldstein et al. 2020). Results were mixed, with only three interventions reporting reductions in depression symptomatology. Of the evidence available, mindfulness-based cognitive behaviour therapy (mCBT) approaches conducted in an individual session with a male facilitator appear to be have the best available evidence and are preferred by fathers themselves (E. E. Cameron et al. 2017; Habib 2012; Livingston et al. 2021). However, the time and resource-intensive nature of such interventions can prove challenging in the perinatal period for fathers, who are typically juggling full-time work alongside care responsibilities. Fathers are a difficult population to engage in mental healthcare (Bateson et al. 2017); men are reluctant to seek help for mental health issues, and are at higher risk of drop-out from treatment than women (Seidler et al. 2016; Spendelow 2015). Further, the prevalence of paternal perinatal mental health difficulties requires a scalable, public health-approach to maximise reach, which may be challenging to achieve through costly, individual, face-to-face therapeutic approaches.

Digital health interventions, such as those delivered electronically using web (eHealth) and mobile (mHealth) applications, may be a promising solution. Emerging evidence shows fathers have higher engagement with digital perinatal support tools than traditional health services (Fletcher et al. 2020; O’Brien et al. 2017), suggesting that mobile tools for mental health support could be a feasible and acceptable option for this population. Explanations for this are limited in the literature, but could potentially be explained by fathers’ preference for self-reliance in seeking information and parenting support (Wade et al. 2022). Cognitive behaviour therapy can be effectively translated to digital platforms for treating maternal postpartum depression, anxiety and stress (Ashford, Olander, and Ayers 2016; Lee et al. 2016; Roman, Constantin, and Bostan 2020). However, very few digital health interventions have been made specifically for fathers, and fewer still have included content targeting fathers’ mental health (Virani, Duffett-Leger, and Letourneau 2019), resulting in limited evidence for the feasibility, acceptability, and effective design of digital perinatal mental health interventions for men.

Further, mHealth apps appear to report consistent usability issues, including in the mental health space. Low engagement and high attrition are increasingly being recognised as a key challenge for digital health interventions, with meta-analytic evidence indicating that approximately half of all participants drop out of app-based interventions for depression (Torous et al. 2020). A recent systematic review identified that only a small fraction of digital health interventions publish the results of usability testing at all, compounding the difficulty of identifying why a digital intervention might be ineffective or encounter high-levels of drop-out (Maramba, Chatterjee, and Newman 2019). Qualitative feedback may provide some insights, with a synthesis of the user experience across 17 mental health apps indicating that the ease of use, usefulness of content and privacy were key barriers for users engaging and maintaining their use of mental health apps (Chan and Honey 2022). Technical issues may also contribute to usability concerns, including errors or bugs in the in the mHealth app, issues with mHealth data integration with other healthcare systems, or issues with internet connectivity in low resource settings (Liew et al. 2019; Meyer et al. 2020). Combined with difficulties engaging fathers in traditional psychological support, a considered approach is warranted in the design of any digital health interventions for supporting fathers’ perinatal mental health.

Therefore, the current study aimed to examine the feasibility, acceptability, and usability of a mobile app-based intervention for paternal mental health in the perinatal period. Evidence-based design principles were developed by synthesising the current literature on supporting men in the perinatal period, which were then used to develop a prototype mobile application. The prototype was evaluated for its usability, acceptability and feasibility with both men experiencing mental health issues in the perinatal period and mental health clinicians. Combined, the results of this study will inform the design of digital mental health support tools for fathers – a population often overlooked and difficult to engage in research and clinical settings alike, for which there is limited evidence to date in the effective design of digital tools.

## 2. Method

### 2.1. Intervention design and development

**Figure 1.**
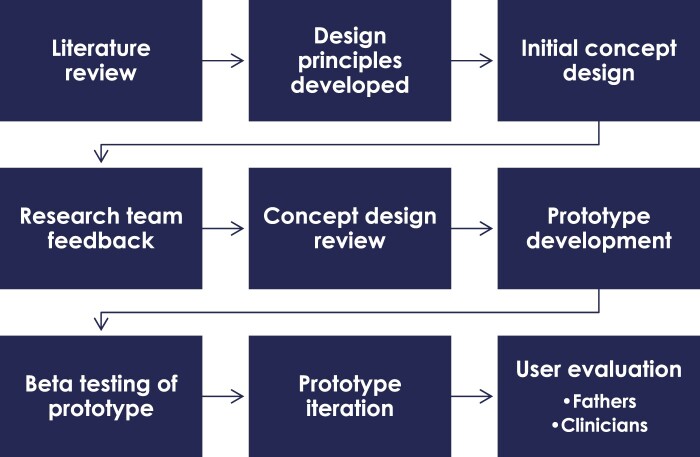
Development process for the Rover app.

The intervention was developed using an iterative, design science approach (Farao et al. 2020). First, design principles were developed by reviewing published literature on effective interventions and methods for engaging fathers in mental health support. A prototype app was then developed using the design principles as a guide, and then evaluated by fathers and clinicians for its usability and feasibility. Details on the generation of design principles and prototype development are outlined in Figure 1 and presented below.

#### 2.1.1. Design principles

Design principles are generalised directives based on experience, examples, or empirical evidence to assist in guiding the design process (Fu, Yang, and Wood 2015). Design principles are typically derived from existing designs or principles presented in the literature, and can range from specific principles, describing single features, to meta-principles, capturing abstract ideas by identifying themes across specific principles (Fu, Yang, and Wood 2015; Kali 2008). For the current study, the literature was searched for design principles from previous interventions targeting fathers’ mental health. Specifically, a search was conducted to identify literature review articles focused on fathers’ mental health intervention, and the reference lists of review articles were manually searched for other relevant literature. The identified literature focused on effective interventions for fathers (Habib 2012; Livingston et al. 2021; O’Brien et al. 2017), fathers’ perinatal needs (Eriksson and Salzmann-Erikson 2013; Fletcher et al. 2008; Salzmann-Erikson and Eriksson 2013; Shatte et al. 2020; Teague and Shatte 2018, 2021), development of web and mobile apps to support fathers (Fletcher et al. 2008; Lee et al. 2016; White et al. 2019), engaging men generally in psychological support (Mahalik et al. 2012; Seidler et al. 2018), and translating clinical psychological practice into digital tools (Huckvale et al. 2020; Schroeder et al. 2020).

Table 1 presents the specific principles generated from the above literature. Specific principles were grouped based on their relevance to the clinical content, visual design, messaging, and app functionality. The specific principles were then synthesised into five broader meta-principles, capturing themes across the clinical, visual, messaging, and functional groups:

1. *Holistic over clinical.* The app should play down its mental health focus and take a holistic perspective to postpartum adjustment.
2. *Collaborative over autocratic.* The app should take an action-oriented, collaborative problem-solving approach with an experienced male facilitator.
3. *Strengthening over modulating.* The app should embrace caring fatherhood as a strength of masculinity.
4. *Adaptive over prescriptive.* The app should be easy to pick up and use around work and family commitments for both in-the-moment and longer-term support.
5. *Fun over serious.* The app should work to build a therapeutic alliance with dads by engaging in informal rapport-building methods.

**Table 1.** Specific design principles used to guide the clinical content, visual design, messaging, and functionality of the prototype app.

*Clinical content*
CBT and/or mindfulness-based Clear clinical pathway for user Goal-oriented Combination of in-the-moment and longer-term support General perinatal support alongside mental health support Flexible delivery, short/concise content
*Visual design*
Analogies with typical male hobbies ‘Disguising’ from being a mental health app
*Messaging*
Action-oriented, positive framing Appearance of male facilitator Warm and humorous tone Informal language/personal anecdotes to normalise common fatherhood challenges Encouragement, advice and confirmation supportive language
*Functionality*
Personalised with names, relevant content Gamification elements Notifications to encourage use

#### 2.1.2. Initial concept design

**Figure 2.**
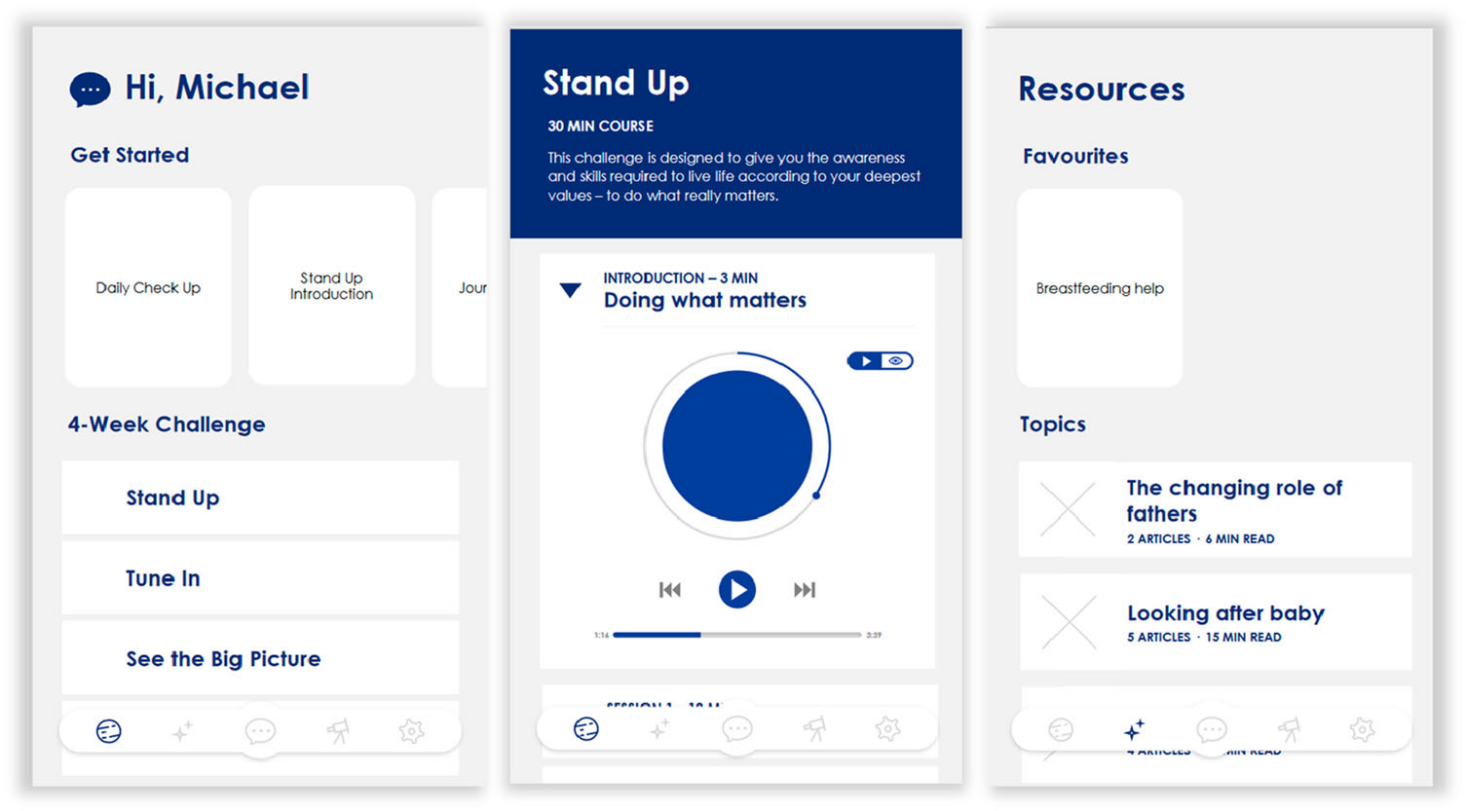
The initial concept design (from left to right): (1) the home page, (2) an introductory audio exercise page for the values module, (3) the resources page.

An initial concept was developed using the above design guidelines (see Figure 2). The self-directed clinical content was based on a previous mHealth app for parents’ mental health (Fuller-Tyszkiewicz et al. 2020), consisting of four modules of audio and interactive exercises designed to help fathers live according to their values, improve their mindfulness, increase positive affect (via gratitude exercises, positive imagery, and cognitive restructuring), and increase their behavioural activity. The initial concept was refined via feedback from the research team, which consisted of a clinical psychologist, developmental psychologist, software engineer, and experts in perinatal mental health and mobile app-based mental health interventions, and a father. Feedback was focused on ensuring the content was relevant and helpful for fathers and on improving the user experience. The initial concept was further refined before development of a prototype app.

#### 2.1.3. Prototype development

**Figure 3.**
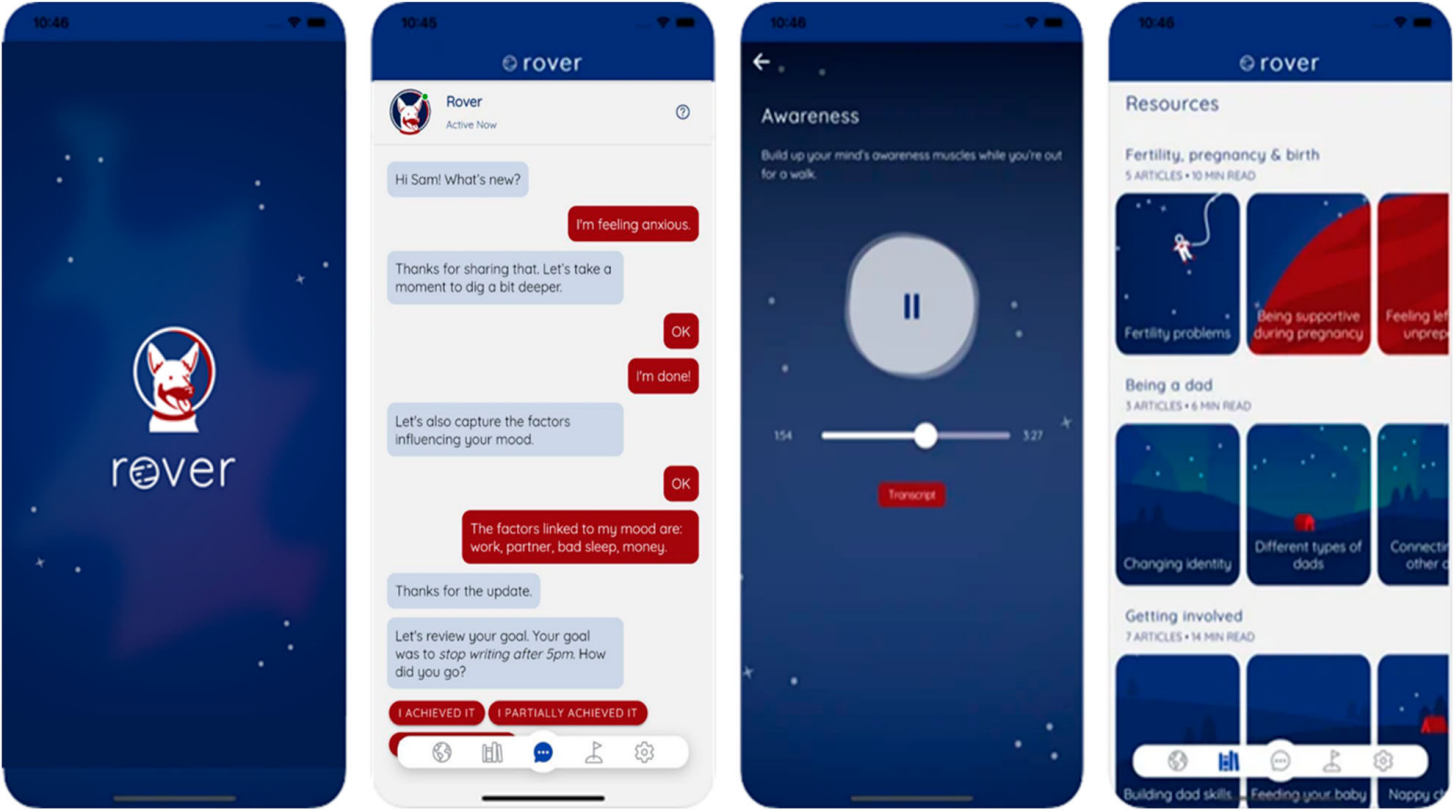
The prototype app pages (from left to right): (1) the splash screen; (2) the chatbot facilitator page; (3) an audio exercise; (4) the resources page.

The prototype app (see Figure 3) was developed by extending the open-source mHealth platform *schema* (Shatte and Teague 2020). A space video game theme was selected for the overall app aesthetic – a typical male hobby popular with new fathers that provided opportunities for integrating humour and analogies into the app messaging and content (Teague and Shatte 2021). The app consisted of five pages: (1) a chatbot facilitator, styled as a dog named Rover, who completed user onboarding and engaged in daily chats such as mood and goal tracking; (2) an exercises page, containing four modules of structured mindfulness-based CBT activities; (3), a resources page, containing 21 brief articles on common challenges for new fathers; (4) a tracking page, supporting user’ self-monitoring of mood and goals, and gamification of exercise completion; and, (5) a settings page, where users could personalise their nickname and notification schedule, and also quickly access contact information for the study team and mental health services.

The clinical content was presented to users as a ‘4-Week MindHack Challenge’ and consisted of four modules: (1) *Plan*, a strengths, values, and goal setting module; (2) *Focus*, a mindfulness module involving self-observation, connecting with the present moment, cognitive diffusion, and acceptance; (3) *Recharge*, a well-being enhancement module using positive psychology techniques and cognitive restructuring; and, (4) *Action*, a behavioural activation module to increase engagement in, and enjoyment of, pleasant or valued activities (see Supplementary Table 1 for further details). Each module consisted of between 30 and 60 minutes of content, and featured self-assessments, audio explanations using a male voice (with transcripts available), exercises, and activities. The modules presented a pathway for users to engage in longer-term support by integrating exercises with explanations and self-assessments. Further, the exercises within modules were brief and flexible in nature, allowing users to access and bookmark favourite exercises for in-the-moment support.

The chatbot, Rover, was designed to appear as a male facilitator partnering with users to complete the intervention. The chatbot was developed using a basic expert systems framework; a type of artificial intelligence that responds to user input by combining the user’s previous responses with conditional rules from a knowledge base (Cameron et al. 2018). The chat flow followed a hierarchical decision tree, with inputs from the user sourced via a menu-based dialogue (similar to Puspitasari et al. 2022). The chatbot dialogue was triggered each time the app was opened or by directly accessing the chatbot page and served several clinical functions (see Supplementary Figure 1). First, the chatbot assisted users in completing a one minute daily depression and anxiety symptom monitoring assessment using the Immediate Mood Scaler (IMS-12) (Nahum et al. 2017), as well as noting what factors may have influenced the current mood (e.g. work, family, sleep, etc.). Users’ daily mood monitoring was tracked and presented as a graph to users on the tracking page. Second, the chatbot also facilitated a weekly goal setting and reviewing activity, where users could set a goal for the week, track their progress using a daily checklist in the tracking page, and review their progress with the chatbot a week later. Third, the chatbot provided recommendations on what exercise the user should complete next by tracking each user’s completion of the four modules, preventing users from losing track of their progress. Finally, the chatbot also supported a structured open chat dialogue, where users could expand on their feelings via an interactive dialogue and be directed to an appropriate exercise based on their recorded daily mood. For further details on the chatbot architecture, refer to Supplementary Figures 2 and 3. Beta testing of the prototype was conducted with members of the research team over a four-week period, focusing on the user experience. Minor usability issues and an additional resource article on fertility complications were addressed prior to a full user evaluation, detailed below.

### 2.2. Participants and recruitment

A user evaluation of the prototype app was conducted with 10 mental health clinicians experienced in working with fathers during the perinatal period, and 43 fathers with pregnant partners or infants aged under 12 months. Clinicians were recruited through an open call to professional groups focused on supporting fathers in allied healthcare. Fathers were recruited via social media advertisements to the platforms Reddit, Facebook and Twitter. Clinicians and fathers completed a brief survey before downloading and using the app for one week and four weeks each, respectively. They then completed a follow-up survey seeking their feedback on the app’s usability. All participants were offered a $50 voucher upon completing the study, and provided informed consent. The study was reviewed and approved by the Deakin University Human Research Ethics Committee 2020-151.

### 2.3. Measures

#### 2.3.1. Demographic questionnaire

Both clinicians and fathers indicated their age, gender, and country of residence. In addition, fathers indicated their education level, infant date of birth, parity status, and prior experience using mental health apps or websites. Further, clinicians were asked to indicate their profession, number of years of experience working with fathers, and their experience using mental health apps both personally and professionally.

#### 2.3.2. Fathers’ mental health

Depression symptoms experienced in the past week were assessed using the Edinburgh Antenatal and Postnatal Depression Scale (EPDS), a 10-item self-report measure designed for perinatal assessment (Cox, Holden, and Sagovsky 1987). Items are rated using a 4-point scale (0–3), and a total score is calculated ranging from 0 to 30 with higher scores indicating more depressive symptoms. Research using the EPDS indicates that a score of ≥9 is optimal for screening moderate to severe depression in fathers (Edmondson et al. 2010; Matthey et al. 2000).

Anxiety and stress symptoms were assessed using the anxiety and stress subscales of the Depression Anxiety Stress Scales (DASS-21) (Lovibond and Lovibond 1996). The DASS-21 anxiety and stress subscales each contain 7 items, with participants rating the frequency of any anxiety and stress symptoms experienced in the past week using a 4-point scale (0–3). Responses are doubled and then summed to form a total score for each subscale (range 0–42), with higher scores indicating more symptomatology. Established cut-off scores for moderate anxiety and stress symptomatology are ≥10 and ≥19, respectively (Lovibond and Lovibond 1996).

#### 2.3.3. App usability

The prototype app’s usability was assessed by both fathers and clinicians using the System Usability Scale (SUS) (Brooke 1996), a 10-item scale of perceived usability. Participants rate the extent to which they agree with each statement using a 5-point scale (from ‘strongly agree’ to ‘strongly disagree’). Total scores are converted to a 0–100 scale, with higher scores indicating higher perceived usability. A score of 68 or more is considered above average usability (Hyzy et al. 2022).

#### 2.3.4. Overall app quality

The overall mHealth app quality was assessed by both fathers and clinicians using the Mobile App Rating Scale (MARS) (Stoyanov et al. 2015). The MARS is a 23-item questionnaire examining the objective quality of mobile health apps using four subscales: (1) engagement, including the app’s interest, interactivity, customisation, and fit to target group; (2) functionality, such as the app’s performance, navigation, and gestural design; (3) aesthetics, including the graphics, layout, and visual appeal; and (4) information quality, covering the quality and quantity of app content. Participants rated each item using a 5-point scale, with higher scores indicating higher quality. Subscale scores are calculated using the mean item score.

#### 2.3.5. App acceptability

The acceptability of the app was assessed using two items from the MARS measure (described above), ‘Would you recommend this app to people who might benefit from it?’ and ‘What is your overall (star) rating of the app?’.

#### 2.3.6. App feasibility

The feasibility of the app as a mental health support tool for fathers was evaluated to determine the degree to which it can be effectively integrated into the usual care workflow (Ng et al. 2019; Park, Nicksic Sigmon, and Boeldt 2022). This assessment was based on usage statistics that tracked the frequency and nature of fathers’ interactions with different features and modules of the intervention, aligning with established practices in digital mental health interventions (Ng et al. 2019; Park, Nicksic Sigmon, and Boeldt 2022). Specifically, we recorded the type, number, and timestamp of interactions over the 4-week period, providing objective measures of frequency, duration, amount, and depth of accessed information at both daily and weekly levels (Perski et al. 2017).

#### 2.3.7. Clinician’s perceived impact

Clinicians were asked to assess the perceived impact of the prototype app using the MARS’s perceived impact scale (Stoyanov et al. 2015). The perceived impact scale consists of six customisable items assessing the extent to which the app is likely to influence the target user’s awareness, knowledge, attitudes, intentions to change, help-seeking, and behaviour change related to the target health behaviour. For the current study, the six items were customised to specify the target users as fathers and health behaviour as perinatal mental health, e.g. ‘Awareness: This app is likely to increase fathers’ awareness of perinatal mental health issues’. Clinicians rated the extent to which they agreed with each item using a 5-point scale (from ‘strongly disagree’ to ‘strongly agree’), with higher scores indicating a stronger perceived impact.

#### 2.3.8. Digital therapeutic alliance

The quality of the relationship felt by the father with the mobile app was assessed using the Mobile Agnew Relationship Measure (mARM) (Berry et al. 2018). The mARM contains 25-items assessing therapeutic relational quality, rated by participants using a 7-point scale (from ‘strongly disagree’ to ‘strongly agree’). Responses are summed to form a total score (range 25–175), with higher scores indicating a higher quality therapeutic relationship. In addition, the mARM contains five subscales calculated using the mean item score: (1) openness, feeling free to make personal disclosures; (2) bond, positive feelings and felt connection towards the app; (3), client initiative, feeling in control and empowered by the app; (4) partnership, feeling in collaboration with the app; and finally (5) confidence, feeling assured in the app’s therapeutic capability.

#### 2.3.9. Qualitative app feedback

Finally, qualitative feedback was elicited using two open-ended questions from the Perceived Usefulness Ease of Use scale (PUEU) (Davis 1989): (1) List the most negative aspects of the app; and (2) List the most positive aspects of the app. The broad nature of the qualitative questions was intended to avoid biasing respondents on what to focus on. Qualitative feedback was collected to illustrate quantitative findings from app usability, quality, perceived impact, and therapeutic alliance measures.

### 2.4. Data analysis

Descriptive statistics were used for quantitative data from the SUS, MARS, and mARM. Results were presented separately for the father and clinician groups. Qualitative and quantitative findings were integrated using a sequential explanatory design – a mixed-methods approach whereby qualitative findings are used to interpret or explain quantitative results (Pluye and Hong 2014).

## 3. Results

### 3.1. Participant characteristics

Table 2 shows the characteristics of the father and clinician samples. The average father was a first-time parent with an infant aged approximately 5 months old, highly educated (82% had bachelor’s degree or higher), and reported elevated depression, anxiety or stress symptoms (60% were above the clinical cut off on any mental health variable). Clinicians were predominantly female psychologists with an average of 8 years of experience working with fathers.

**Table 2.** Characteristics of the father and clinician samples.

Variable	Mean/n	SD/%	Range
**Fathers**			
Age	31.23	(4.09)	24–39
Sex (male)	43	(100%)	
Country			
Australia	8	(19%)	
Canada	5	(12%)	
United Kingdom	17	(40%)	
United States	13	(30%)	
Education Level			
Postgraduate	14	(33%)	
Bachelor	21	(49%)	
Trade certificate	7	(16%)	
None of the above	1	(2%)	
Perinatal Period			
Pregnancy	8	(19%)	
Postpartum	35	(81%)	
Infant age (months)	4.94	(5.16)	−6.69–12.16
First-time father [parity] (%)	26	(60%)	
Used mental health app/website before (Yes)	9	(21%)	
EPDS	10.56	(5.48)	1–22
DASS-21 Anxiety	6.60	(6.58)	0–28
DASS-21 Stress	17.86	(9.00)	0–36
**Clinicians**			
Age	40.8	(10.86)	27–60
Sex (male)	1	(10%)	
Country (Australia)	10	(100%)	
Profession			
Psychologist	6	(60%)	
Psychiatrist	1	(10%)	
Social worker	1	(10%)	
Counsellor	1	(10%)	
Other	1	(10%)	
No. years clinical experience	7.93	(8.41)	0.5–25
Mental health app experience			
Personally use apps/wearables	7	(70%)	
Professionally use apps/wearables	5	(50%)	

### 3.2. Participant evaluation findings

Quantitative findings (shown in Table 3) are discussed for the app’s usability, quality, content, and therapeutic relationship below. Findings are illustrated and expanded using qualitative feedback from participants in Table 4.

**Table 3.** Descriptive statistics from the usability, quality, perceived impact, and therapeutic relationship measures.

	Fathers			Clinicians		
	Mean	SD	Range	Mean	SD	Range
SUS	75.09	(14.52)	42.5–100	83.25	(12.8)	65–97.5
MARS						
Engagement	3.47	(0.58)	2.25–4.67	4.23	(0.49)	3.2–4.6
Functionality	4.07	(0.60)	2.75–5	4.48	(0.48)	3.75–5
Aesthetics	3.88	(0.76)	1.67–5	4.13	(0.67)	3.33–5
Information	3.75	(0.56)	2.67–5	4.30	(0.53)	3.33–5
Overall Quality	3.79	(0.39)	2.98–4.63	4.28	(0.46)	3.47–4.84
Recommend app	3.19	(0.96)	1–5	4.20	(1.03)	2–5
Star Rating	3.63	(0.72)	2–5	4.00	(0.47)	3–5
Awareness	–	–	–	4.70	(0.48)	4–5
Knowledge	–	–	–	4.60	(0.70)	3–5
Attitudes	–	–	–	4.30	(0.95)	2–5
Intention to change	–	–	–	4.30	(0.82)	3–5
Help seeking	–	–	–	4.30	(0.82)	3–5
Behaviour change	–	–	–	4.40	(0.70)	3–5
mARM						
Openness	4.74	(1.14)	2.25–6.5	–	–	–
Bond	5.08	(0.92)	2.4–6.4	–	–	–
Client initiative	4.68	(0.87)	3–6.5	–	–	–
Confidence	4.75	(1.12)	1.86–6.86	–	–	–
Partnership	4.89	(1.03)	2–7	–	–	–
Mean score	4.79	(0.89)	2.72–6.44	–	–	–
Total	119.64	(22.21)	68–161	–	–	–

**Table 4.** Themes and exemplar quotes from the qualitative feedback.

Theme	Exemplar Quote
Usability	‘Simple, easy and clear design. I enjoyed using it.’ Father (Pregnancy) ‘It was fluid, intuitive, robust and worked really professionally.’ Father (Pregnancy) ‘On the main (globe icon) page I sometimes couldn't work out if there were more subjects I had to complete as it was a little busy.’ Father (Postpartum) ‘I would like to see the graphs showing your current stages (levels of anxiety/depression) organized more closely to the goal setting screen. All of these progress monitoring elements should be closer together.’ Father (Postpartum)
Quality	‘The look and design of the app is beautiful, very engaging.’ Clinician (Psychologist) ‘I found it quite simple to use, to have very evidenced and clearly communicated based psycho-education and easily implementable exercises to improve a father's mental health.’ Clinician (Psychologist) ‘Is it trying to be too many things to too many people? Would dads be willing to answer a quick questionnaire before using the app regarding their priorities – what they want out of the app – to better customise the offerings?’ Clinician (Psychiatrist) ‘Very functional, perfect presentation etc. [But] I don't think the app would be useful to people who don't enjoy writing things’ Father (Postpartum)
Perceived Impact	‘The mindfulness meditation exercises were relatively short and there is a range of formal and informal practices. This is good for fathers as entering into parenthood is a busy time. Having short and both informal and formal exercises increases the likelihood of engagement and success.’ Clinician (Psychologist) ‘The daily check in is guided in such a way that makes you really analyze how you are feeling without taking much time or effort.’ Father (Postpartum) ‘Overall, the concept of mindfulness, which I've never tried before, actually had an effect I think.’ Father (Postpartum) ‘With lock down coming into effect and becoming the primary carer for my infant – I had very little time to use it and it could have had a setting to ditch the AI and focus on the lessons and plans.’ Father (Postpartum)
Digital therapeutic alliance	‘Informed, caring approach that I would certainly find helpful if I was an isolated struggling dad. I wish it had been there when I had my first child more than a decade ago!’ Clinician (Psychiatrist) ‘I just wanted to say I found it very useful, as an ex-sufferer from depression from my when my first child was born 3.5 years ago, I wish I had access to this sort of platform back then, especially with having something to talk to.’ Father (Postpartum) ‘The text conversation format of the communication felt a bit fake, pretending to be a person when it's clearly not.’ Clinician (Psychiatrist) ‘It's hard to “open up” to an app, especially without knowing who might end up seeing your responses.’ Father (Postpartum)

#### 3.2.1. Usability

The SUS mean scores for both fathers and clinicians were higher than the threshold for acceptable usability, with clinicians rating the app higher than fathers (see Table 3). Qualitative feedback consistently indicated that the app was simple and easy to use by both fathers and clinicians. Usability strengths reported by participants were the daily notifications to prompt use and the availability of transcripts in the audio exercises.

Several usability issues were also raised, including narrow visual analogue scales (VAS) making selections difficult on narrow phone screens and an overcrowded layout on the home screen.

One participant also suggested redesigning the *Tracking* tab to integrate the three tracking elements together: namely, the usage, mood and goal tracking elements. Such a layout may offer users new insights into how these factors interrelate; for example, depicting associations between user’s mental health and engagement in new habits.

#### 3.2.2. Quality

Participants rated the *overall quality* of the app highly (*M* = 3.9, *SD* = 0.44), with clinicians again rating the app higher than fathers (see Table 3). Fathers gave the highest scores for the app’s *functionality* and *aesthetics*, particularly for the app’s performance (*M* = 4.19, *SD* = 0.82) and layout (*M* = 4.02, *SD* = 0.74). Fathers gave the lowest scores for the app’s *engagement*, particularly for customisation (*M* = 3.14, *SD* = 0.8). This was reflected in their qualitative feedback, with fathers requesting for customisation capabilities across the breadth of the app’s features, including in treatment plans, resources, mood tracking, chatbot discussions, and overall theme. Qualitative feedback from a clinician also noted that enabling more customisability to tailor content to fathers’ specific needs could be beneficial.

Overall, clinicians gave very high scores for all subscales of the MARS, with mean scores above 4 across all items. Highest scores were given for the app’s *functionality* and *information*, particularly for the app’s performance and gestural design (*M* = 4.6, *SD* *=* 0.52 for both), and the app’s information quality and visual information (*M* = 4.5, *SD* *=* 0.71; *M* = 4.5, *SD* = 0.53, respectively). Qualitative feedback from clinicians showed appreciation for the use of ACT and CBT principles delivered in brief exercises, noting that this would be useful for integrating the app within their clinical practice.

#### 3.2.3. Acceptability

The overall star rating provided by participants was 3.63 (*SD* = 0.73), with clinicians providing a higher star rating than fathers (see Table 3). Both fathers and clinicians indicated that they would be willing to recommend the app to others, with mean scores indicating that fathers would recommend the app to ‘several people’ while clinicians would recommend the app to ‘many people’. One father who indicated that they would not recommend the app to others clarified in the qualitative feedback that the app’s use of written interactivity was a hindrance. A similar consideration was noted by one clinician in the qualitative feedback, with written exercises potentially feeling like ‘homework’ for fathers balancing work and family commitments.

#### 3.2.4. Feasibility

Over the four-week duration of the intervention, father participants interacted with the Rover app an average of 8.67 times a day for an average duration of 5.41 min. (*SD* = 9.23, *SD* = 9.38, respectively), with the average total use being 151.55 min. (*SD* = 262.7). In week 1, daily interactions averaged 14.67 (*SD* = 13.4) with 90.67% of participants accessing the app every day; by week 4, daily interactions averaged 8.12 (*SD* = 13.9) with 58% of participants continuing to interact with the app every day. The chat function was the most intensively used feature of the app, with an average of 82.19 min. (*SD* = 183.04) over 118.37 interactions (*SD* = 116.03). The resources section was the least used feature of the app, with an average of 0.92 min. (*SD* = 3.08) over 6.67 interactions (*SD* = 13.07).

#### 3.2.5. Perceived impact

Clinicians also rated the app very highly on the MARS for its ability to improve fathers’ *awareness*, *knowledge*, *attitudes*, *intention to change*, *help seeking*, and *behaviour change* regarding their perinatal mental health (*M*’s ranging from 4.3–4.7, see Table 3). Clinicians’ qualitative feedback highlighted the app’s potential to normalise mental health experiences in the perinatal period for fathers, with one clinician requesting that the app also integrate further normalising of fathers’ seeking more formal support. Clinicians also noted specific features of the app that were likely to encourage fathers’ uptake and continued engagement with the app, including having a range of brief formal and informal exercises, and easy tracking of behaviour change.

In their qualitative feedback, fathers noted that they felt the app had had an impact on them, with many noting that they experienced improved awareness of their moods and mental states. Fathers appeared to particularly appreciate the mood tracking, goal tracking, and mindfulness exercises. One barrier raised by both a clinician and father in qualitative feedback was the daily chatbot interaction potentially impeding fathers’ ability to quickly receive support when needed, suggesting that a ‘skip’ function be added to the AI component.

#### 3.2.6. Digital therapeutic alliance

Overall, fathers rated the app moderately for the perceived digital therapeutic alliance using the mARM (see Table 3). Highest scores were given for the *bond*, particularly for items regarding feeling friendly towards and accepted by the app (*M* = 5.4, *SD* = 1.14; *M* = 5.79, *SD* *=* 0.94, respectively). A comparison between first-time and experienced fathers indicated that first-time fathers felt a higher quality therapeutic relationship with the app than experienced fathers (first time fathers: *M* = 124.41, *SD* = 20.1; experienced fathers: *M* = 112.35, *SD* = 23.87; *t*(41) = 1.78, *p* = .04), with this being the only difference between groups across measures (see Supplementary Table 2). Lowest scores on the mARM were given for the *openness* subscale, particularly for items regarding holding back important things and sharing sensitive personal matters with the app which were both rated moderately (*M* = 4.33, *SD* = 1.64; *M* = 4.44, *SD* *=* 1.30).

Qualitative feedback from experienced fathers and the clinician who was also a father indicated that the app would have been helpful for them personally the first time around. These participants particularly highlighted the app’s ability to help them feel socially connected and supported during a difficult time. Fathers qualitative feedback indicated that the app came across as friendly and helpful, with many mentioning they formed a bond with the chatbot AI feature specifically. Fathers particularly appreciated having the chatbot checking in on them, with some noting that they felt the dog AI was a companion they could rely on to talk to and share their feelings with. One father particularly liked the open chat dialogue, which encouraged users to dig deeper in sharing their feelings via an interactive dialogue. While most fathers indicated that they enjoyed the chatbot feature, one clinician raised that the chatbot could potentially feel inauthentic in their qualitative feedback. Beyond the chatbot, qualitative feedback from both fathers and clinicians also indicated that the male narrator voice used in audio explanations and exercises helped in forming a bond to the app. Both groups mentioned that the voice was calming, and one father appreciated the Australian accent.

## 4. Discussion

This study aimed to examine the feasibility, acceptability, and usability of an app-based intervention developed for paternal mental health in the perinatal period. Following best practice, we developed a set of specific and meta-design principles guided by the literature, iteratively designed an app using feedback from a team of multidisciplinary experts, developed a prototype mHealth app, and sought user feedback from clinicians and fathers. Overall, the prototype app demonstrated good usage, usability, and quality, as well as high levels of perceived impact and moderate levels of therapeutic alliance, as rated by objective measures. The acceptability and feasibility of the app was moderate-to-high, with fathers and clinicians alike agreeing that they would recommend the app to others in need and just over half of fathers continuing to use the app every day across the four-week study duration. Qualitative feedback helped clarify the prototype app’s strengths and weaknesses, including both human–computer interaction and clinical features. This research has important implications for future studies aiming to engage fathers in digital mental health initiatives – an overlooked and difficult to reach target group – as well as broader implications for user-centred design of mHealth applications.

First, the design principles specified that the app’s clinical content should be a flexibly-delivered, brief, mindfulness-based CBT approach with a combination of in-the-moment and longer-term support. Results showed that fathers and clinicians alike were favourable of the app’s clinical content, particularly the mindfulness exercises, and mood and goal tracking elements. Other clinical content that involved more writing, such as the *Recharge* and *Action* modules, were less popular, potentially due to the time burden required to complete them. The design guidelines also specified including general perinatal support beyond mental health in the app content, which was delivered in the prototype via the *Resources* page. These additional resources were appreciated by fathers and clinicians for their brief, evidence-based content, and links to more detailed information, however feasibility data indicated this was rarely accessed. Importantly, the clinical content could be improved by enabling greater personalisation of both the treatment programme and resources for fathers. This could include tailoring the content to fathers via parity status, infant age, and severity of depression and anxiety symptoms. Such feedback is commonly reported in app-based interventions more broadly (Oakley-Girvan et al. 2021), and mental health apps specifically (Mayer et al. 2022; Polhemus et al. 2022), indicating that users expect more opportunity to tailor their experience and lead the management of their intervention plan. Fathers’ desire for more customisation in the app’s clinical content could indicate that the meta-design principle ‘collaborative over autocratic’, whereby the app should partner with fathers to lead their own treatment plan, was not sufficiently met in the prototype app design. While some level flexibility and customisation was provided, it is clear that creating more opportunities for men to personalise their experience may encourage greater adherence and retention to the app-based intervention, and in turn, may also strengthen the therapeutic partnership felt between the father and mHealth application (Tong et al. 2022).

Second, the design principles specified that the app’s visual design should disguise it from being a mental health app using analogies with typical male hobbies. This was achieved in the prototype by using a space video game theme across all elements, including the chatbot, clinical exercises, and resources. Clinicians and fathers’ alike rated the app’s aesthetics very highly, and qualitative feedback from both groups consistently focused on the strength of the app’s visual design and layout. Importantly, these design features were carefully considered given background research on the target population, with prior app-based interventions targeting adults with depression reporting participants’ strongly disliked ‘child-like’ graphics such as emoticons in mood trackers (Mayer et al. 2022). Further, feedback from one father indicated that the visual design could be improved by enhancing the progress monitoring elements provided in the *Tracking* page. Future iterations could consider refining this visual feedback, which could potentially support successful behaviour change by enabling greater insights into how a user’s moods and habits are interrelated. Such work should consider best practices around the collection, reflection, and communication of data, with prior research on mental health apps demonstrating these are typically not adhered to (Cho et al. 2022; Devakumar et al. 2021; Polhemus et al. 2022).

Third, the design principles required the app’s messaging to be warm and humorous, action-oriented, and delivered by a male facilitator. This was achieved in the prototype by using a male voice for the audio elements (including explanations and exercises) and developing a chatbot AI styled as a dog named Rover. Fathers rated the app highly for the felt bond to the app, particularly first-time fathers, and qualitative feedback indicated that they found the app helpful, supportive, and friendly. These findings support prior work on using chatbots for mental health, where users typically rate the chatbot highly for empathy despite clinicians noting that such systems fall short of true empathetic care (Abd-alrazaq et al. 2019; Boucher et al. 2021). Clinicians and fathers particularly liked the male Australian voice used in audio elements, noting the voice was clear, calm, and made the experience of using the app more personal to the study’s target audience. Notably, fathers and clinicians expressed in the qualitative feedback that the Rover chatbot could be improved by creating more variance in its dialogue and enabling the ability to ‘turn off’ the chatbot as an app setting. The chatbot was created using a basic expert systems framework, with most inputs following structured options and only limited opportunities for users to engage freely in open dialogue conversations. This design decision was made given the high-risk mental health use case of the chatbot application, with stricter conditional rules reducing the likelihood of any unforeseen issues occurring in an AI system with greater autonomy (Miner, Milstein, and Hancock 2017; Shatte, Hutchinson, and Teague 2019). Future work could consider developing effective responsible AI protocols for mental health chatbots that balance the need for human-like adaptive responses with the risks associated with clinical settings. Such systems could use natural language processing trained using existing texts available from online chat-based support groups (D’Alfonso 2020), which are popular with fathers and demonstrate a range of supportive behaviours (Teague and Shatte 2021).

Finally, the design principles developed from previous literature stated that the app’s functionality should encourage engagement using gamification strategies and notifications and allow users to personalise the app with their name. Participants rated the app very highly for its functionality on the MARS, covering the app’s performance, navigation, and gestural design, and its usability on the SUS, which included the app’s flexibility, learnability, operability, and satisfaction. Qualitative feedback indicated that participants were satisfied with the app’s functionality, particularly the simple design and use of notifications, but were seeking more customisation around the notification schedule. Further, future work should consider extending design principles around app functionality to include consideration of data sharing and privacy policies. While the prototype included a privacy policy and detailed information on data access and sharing in the app store description, fathers gave moderate scores on the mARM *Openness* subscale and qualitative feedback from fathers indicated some concerns around data privacy. Such concerns about the open disclosure of personal information with the prototype app likely reflect the widespread prevalence of mHealth apps sharing data with third parties for commercial advertising purposes, and use of misleading information in app store descriptions around the validity of the app content and privacy practices (Huckvale et al. 2020; Larsen et al. 2019). Future work could address this by including a brief, lay description of the app’s privacy policy as part of the onboarding process to allay fathers’ concerns around disclosure of sensitive personal information. This should include information regarding the types of data that are collected, where the data are stored and shared with, data encryption practices, and explicit statements about potential selling of data to third party companies.

Combined, the results indicate that mobile apps created using our design guidelines are a feasible and acceptable way for fathers to access and engage with perinatal mental health support. The convenience and accessibility of such digital tools offer fathers the ability to overcome barriers commonly reported by men in accessing mental healthcare, including stigma, time constraints, and difficulty in accessing appropriate resources (Bateson et al. 2017). Given the popularity of daily mood monitoring amongst participants, the app has particular implications for scalable early identification and preventative support (Golubnitschaja, Kinkorova, and Costigliola 2014). Further research is needed to examine the efficacy of the intervention prior to any implementation in clinical practice. Usability researchers could investigate novel ways to support both mothers and fathers’ perinatal mental health through mHealth interventions. Partner support is recognised as one of the strongest modifiers of perinatal mental health in both parents (Cluxton-Keller and Bruce 2018; Pilkington et al. 2015), with such a tool requiring consideration of both individual-level and couple-level support.

While the current study has several strengths, including its detailed design and development process and evaluation with a group of 53 users of both fathers and clinicians, there are several important limitations that should be considered when interpreting the results. First, this study did not include an evaluation of the intervention’s effectiveness. An effectiveness evaluation is currently being undertaken as part of a broader randomised controlled trial. Second, the trial was conducted with fathers in English-speaking, high-income countries recruited using online social media platforms during the COVID-19 pandemic period, and thus may not generalise to fathers in other cultures or contexts, or with low access to digital platforms. Clinicians that provided feedback were mostly female, reflecting real-world gender differences in the Australian mental health workforce (Australian Institute of Health and Welfare 2019). Future work could consider investigating whether clinical insights differ on father’s mental health interventions based on clinician gender. Finally, the qualitative feedback was collected via survey using two open-ended questions regarding the most positive and negative aspects of the app. Thus, the qualitative data were not intended to achieve full representation of participants’ nuanced considerations of the app’s usability. Future work could consider a more in-depth qualitative study with users to garner further thematic depth in participant’s subjective experiences using app-based interventions.

## 5. Conclusion

In conclusion, the current study utilised best practices to develop an app-based intervention for fathers’ perinatal mental health and evaluate its feasibility and usability. Specific and meta design principles were generated from the available literature, which informed the development of a prototype intervention. Evaluations from fathers and mental health clinicians indicated that the prototype intervention was acceptable, including its clinical content, visual design, and functionality. Future versions of the app should aim to improve the customisation and personalisation of the clinical and psychoeducational content to the user’s unique context. To our knowledge, this work is the first perinatal mental health app designed specifically for men, with results providing important guidance to clinicians and researchers alike on designing high quality digital health initiatives for fathers.

## Supplementary Material

Supplementary.docx
