# Supplementary material for: User-centred design and evaluation of an mHealth app for fathers’ perinatal mental health: a feasibility, acceptability, and usability study
Source: Behav Inf Technol. 2025 May 9;44(20):4950–64. doi: 10.1080/0144929X.2025.2502474 (PMC12080457; doi:10.1080/0144929X.2025.2502474)
Supplement: Supplementary.docx [file TBIT_A_2502474_SM3751.docx]

Supplementary

Table 1

Clinical content of the app

| Module | **Description** |
| --- | --- |
| 1. Plan | Recognizing personal strengths and qualities, identifying fundamental values in various areas of life (such as work, relationships, leisure, and health), assessing how well one's current life aligns with those values, differentiating between values and goals, and establishing goals based on values. |
| 1. Focus | Enhancing mindful awareness by observing sensations, thoughts, and emotions without judgment or the need to modify their frequency and nature. Engaging in mindful breathing, walking, and photography, and progressive muscle relaxation exercises. |
| 1. Recharge | Applying positive psychology strategies, such as keeping gratitude journals, visualizing positive experiences, and savouring moments of joy to enhance positive emotions. Employing cognitive restructuring techniques to diminish negative emotions and boost self-esteem and optimism. |
| 1. Action | Behaviour activation exercises, including tracking daily activities related to relationships, work, leisure, and health, while increasing engagement in enjoyable or meaningful activities. |

Table 2

Comparison between first-time (*n*=26) and experienced (*n*=17) fathers across measures

|  | First-time fathers | | | | Experienced fathers | | | |  | |  | |
| --- | --- | --- | --- | --- | --- | --- | --- | --- | --- | --- | --- | --- |
|  | Mean | SD | Min-Max | Mean | | SD | Min-Max | *t* | | *p* | |  |
| SUS | 74.57 | (15.41) | 42.5-100 | 75.88 | | (13.46) | 50-97.5 | -.29 | | .39 | |  |
| MARS |  |  |  |  | |  |  |  | |  | |  |
| Engagement | 3.56 | (0.57) | 2.25-4.7 | 3.32 | | (0.58) | 2.5-4.5 | 1.34 | | .09 | |  |
| Functionality | 4.06 | (0.62) | 2.75-5 | 4.09 | | (0.59) | 3-5 | -.16 | | .44 | |  |
| Aesthetics | 3.81 | (0.83) | 1.67-5 | 4.00 | | (0.64) | 3-5 | -.81 | | .21 | |  |
| Information | 3.71 | (0.57) | 2.67-5 | 3.80 | | (0.56) | 2.67-4.67 | -.51 | | .31 | |  |
| Overall Quality | 3.78 | (0.43) | 2.98-4.63 | 3.80 | | (0.34) | 3.08-4.35 | -.15 | | .44 | |  |
| Recommend app | 3.12 | (0.91) | 1.25-4.25 | 3.29 | | (1.05) | 1.5-4 | -.57 | | .28 | |  |
| Star Rating | 3.50 | (0.71) | 2.67-4.62 | 3.82 | | (0.73) | 2.86-4.52 | -1.45 | | .08 | |  |
| mARM |  |  |  |  | |  |  |  | |  | |  |
| Openness | 4.61 | (1.11) | 2.25-6.5 | 4.93 | | (1.18) | 2.75-6.5 | -.88 | | .19 | |  |
| Bond | 4.98 | (0.96) | 2.4-6.4 | 5.25 | | (0.85) | 3.4-6.4 | -.94 | | .18 | |  |
| Client initiative | 4.63 | (0.92) | 5-6.5 | 4.75 | | (0.81) | 3.5-6 | -.45 | | .33 | |  |
| Confidence | 4.56 | (1.16) | 1.86-6.86 | 5.06 | | (1.01) | 2.86-6.43 | -1.46 | | .08 | |  |
| Partnership | 4.75 | (1.01) | 2-6.67 | 5.12 | | (1.06) | 3.33-7 | -1.16 | | .13 | |  |
| Mean score | 4.97 | (0.80) | 3.44-6.44 | 4.49 | | (0.95) | 2.72-6.04 | 1.78 | | .04* | |  |
| Total | 124.41 | (20.10) | 86-161 | 112.35 | | (23.87) | 68-151 | 1.78 | | .04* | |  |

Figure 1

Data flow diagram at level 0


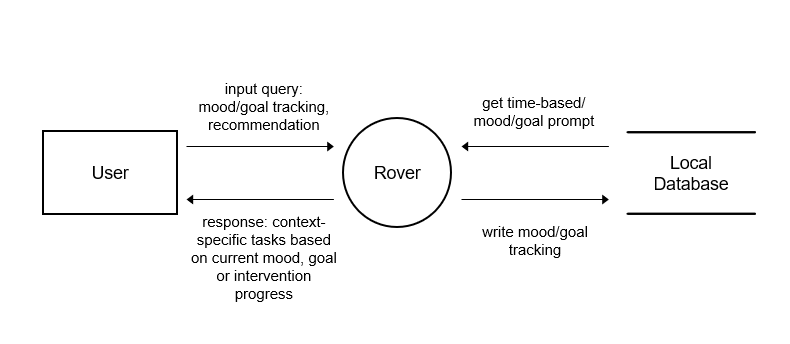


When the user selects an input query (i.e., mood tracking, goal tracking or recommendation), the app queries a local database to determine which prompts to display to the user. For example, if the user selects mood tracking, the app will query the database for mood tracking prompts and record data from the user (e.g., current mood ratings). If the user selects goal tracking, the app will get the user’s current goal from the database and prompt the user to provide an update on their progress (i.e., complete or incomplete), and then ask the user to input a new goal. Finally, if the user selects to be recommended which task to complete next, the app will query the local database on the user’s progress or recent mood tracking inputs, and respond with a link to the recommended task.

Figure 2

Rover chatbot expert system


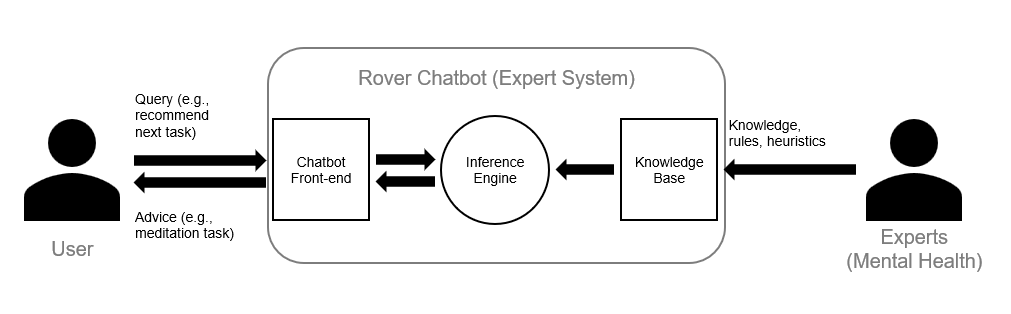


The chatbot system of the Rover app is built as an expert system. Users provide input queries through the chatbot frontend interface, through predefined or structured choices delivered via a menu-based decision tree framework (e.g., selecting mood from a list of moods and then using sliders for ratings). This information is passed into the inference engine, which determines the context of the input (e.g., sad or happy) and then matches appropriate tasks for the user to complete from the knowledge base. The knowledge base is built on information provided by mental health experts and is matched to the user’s context based on their prior inputs and predefined rules.

Figure 3

Menu-based decision-tree framework


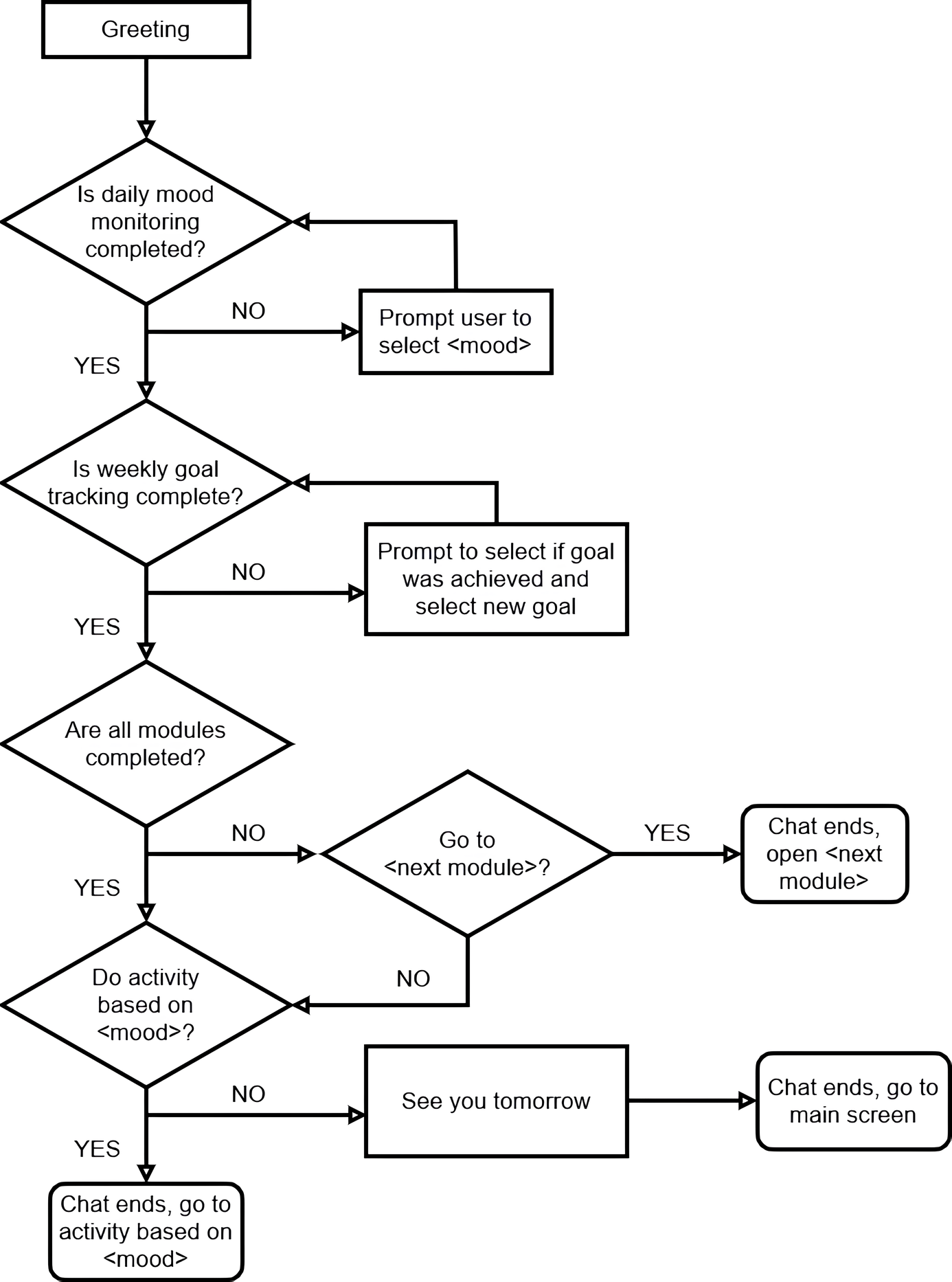


The chat dialogue is structured using a menu-based decision tree with hierarchical rules. When the user opens the app, the chatbot follows the decision tree to ensure that important tasks such as mood monitoring and goal tracking are completed, and to prompt the user to complete the next module in the intervention or do an activity based on their previously reported mood.
